# Creative Hobbies as a Protective Factor Against Stress During the COVID-19 Pandemic in Older Adults

**DOI:** 10.1093/geroni/igab046.051

**Published:** 2021-12-17

**Authors:** Sarah Israel, Darby Mackenstadt, Carolyn Adams-Price

**Affiliations:** 1 Mississippi State University, Mississippi State University, Mississippi, United States; 2 Mississippi State University, Starkville, Mississippi, United States

## Abstract

The COVID-19 pandemic dramatically impacted our way of life, leading to increased rates of anxiety and depression (Panchal et al., 2021). The implications may be worse for older adults who account for 80% of all COVID deaths (Freed et al., 2020). Meanwhile, prior to the pandemic, Adams-Price and colleagues (2018) found that creative hobby participation provided slightly different benefits for middle-aged and older adults. Specifically, evidence suggested that middle-aged adults may use their creative hobby more for stress relief than older adults. Using a sample of 239 women, aged 40 to 84 years old (M = 59.7), we examined whether the degree to which viewing one’s creative hobby as a component of one’s identity related to perceived stress, health anxiety, and depressive symptoms. In addition, we wanted to know whether these relationships were moderated by age. Single moderation models suggest that viewing one’s creative hobby as a part of their identity was related to higher health anxiety and reporting more depressive symptoms. In addition, age was related to reporting lower perceived stress, health anxiety, and depressive symptoms. Lastly, age provided a significant moderation effect to the relationship between degree of identity associated with one’s creative hobby and perceived stress such that middle-aged adults with a high degree of identification with their creative hobby reported the most perceived stress while older adults with a high degree of identification with their creative hobby reported the least perceived stress. Implications for older adult’s well-being and adaptiveness to the COVID-19 pandemic will be discussed.

